# NeuroManager: a workflow analysis based simulation management engine for computational neuroscience

**DOI:** 10.3389/fninf.2015.00024

**Published:** 2015-10-13

**Authors:** David B. Stockton, Fidel Santamaria

**Affiliations:** ^1^Biomedical Engineering Program, The University of Texas at San AntonioSan Antonio, TX, USA; ^2^UTSA Neurosciences Institute, The University of Texas at San AntonioSan Antonio, TX, USA

**Keywords:** grid computing, NEURON, NeuroML, simulation, parameter search, MATLAB

## Abstract

We developed NeuroManager, an object-oriented simulation management software engine for computational neuroscience. NeuroManager automates the workflow of simulation job submissions when using heterogeneous computational resources, simulators, and simulation tasks. The object-oriented approach (1) provides flexibility to adapt to a variety of neuroscience simulators, (2) simplifies the use of heterogeneous computational resources, from desktops to super computer clusters, and (3) improves tracking of simulator/simulation evolution. We implemented NeuroManager in MATLAB, a widely used engineering and scientific language, for its signal and image processing tools, prevalence in electrophysiology analysis, and increasing use in college Biology education. To design and develop NeuroManager we analyzed the workflow of simulation submission for a variety of simulators, operating systems, and computational resources, including the handling of input parameters, data, models, results, and analyses. This resulted in 22 stages of simulation submission workflow. The software incorporates progress notification, automatic organization, labeling, and time-stamping of data and results, and integrated access to MATLAB's analysis and visualization tools. NeuroManager provides users with the tools to automate daily tasks, and assists principal investigators in tracking and recreating the evolution of research projects performed by multiple people. Overall, NeuroManager provides the infrastructure needed to improve workflow, manage multiple simultaneous simulations, and maintain provenance of the potentially large amounts of data produced during the course of a research project.

## Introduction

Access to High Performance Computing (HPC) resources allows exploring the parameter space of complex neurobiological models. As the number of parameters increases it is necessary to keep track of the provenance of simulator configuration, reference models and implementations, data produced by the simulations, the analyses performed and their results; it is also important that the software itself be well documented, tested, and tracked (Gewaltig and Cannon, 2014). Such activities become more cumbersome when using heterogeneous computational infrastructure to perform multiple simulations in parallel (Casanova et al., 2004). By analyzing the workflow of these processes, it is possible to automate them and increase throughput while minimizing delays, errors, and loss of data. Thus, it is important to develop workflow automation tools to set up simulations, label data files, and track analyses to increase productivity and reproducibility of computational neuroscience research.

We have developed a software tool we call NeuroManager to organize modeling efforts using different simulators and computing infrastructure. NeuroManager is based on an analysis of the simulation submission workflow for several neuroscience simulators. NeuroManager is written in object-oriented MATLAB (Natick, MA), a widely used numerical analysis and visualization software suite that is used extensively in engineering and neurophysiology (Drongelen, 2007; Cui et al., 2008; Gabbiani and Cox, 2010; Van Drongelen, 2010; Cohen, 2014; Wallisch, 2014) and is increasingly part of biology education (Gross, 2004; Stefan et al., 2015). NeuroManager virtualizes the hardware, the user, and the simulator. The objects in NeuroManager can be used to generate Machine Sets composed of heterogeneous computational resources, from desktops to HPC centers. The simulator also becomes an object that combines with the specific simulation files, thus allowing the tracking of the evolution of simulation-simulator during a project. Finally, the steps needed for a user to interact with different systems, for example the different steps necessary to submit a simulation run in a local server or a cluster, are also virtualized. The object-oriented approach allows the generation of object trees that can help in keeping the provenance of simulators, data, and their analyses. The software provides progress notification, automatic organization, and labeling of data and results, and integrated access to MATLAB's analysis and visualization tools. The program generates compiled MATLAB code to distribute across platforms, minimizing the number of licenses required. Since our focus is neuroscience, we have developed our code to support a variety of standard neuroscience simulators. Altogether NeuroManager provides a unified platform that reduces the complexity of developing and analyzing computational projects.

The intended users of our software are laboratories that have a need to keep computational simulation sessions and their evolution organized, efficiently use heterogeneous computational resources, and preserve the provenance of simulations and their analyses across multiple users. However, our software can also be used for teaching. While this paper presents the motivation, theory, and design of the workflow and of the software, the Supplemental Materials provides a detailed description of each workflow step. In addition, we provide the code and an extensive User Guide with examples at the GitHub site mentioned below.

## Workflow of simulation submissions in computational neuroscience

A workflow is an abstraction of the set of tasks required to run a simulation (Garijo et al., 2014). In general, a simulation-analysis task requires breaking down the process into workflow stages (Deelman et al., 2009). We developed a list of abstract workflow stages by analyzing the submission of simulation jobs using computational neuroscience tasks and tools. In general we assumed that a user operates a **host** computer to run a simulation on a **remote** computer or cluster, on which a software simulator is installed. For our analysis we used a combination of two types of host computers: Windows and UNIX; three simulators: MATLAB-only code, NEURON (Hines and Carnevale, 2001), and MCell (Stiles and Bartol, 2000); and three remote machine types: Linux multi-core server, Sun Grid Engine (SGE) Cluster, also known as Univa Grid Engine (Univa, 2015), and Simple Linux Utility for Resource Management (SLURM) Cluster (SLURM, 2015). We identified nine abstract stages:

1. General setup on host and remote resources
2. Build simulators on remotes
3. Upload model files to remotes
4. Fetch simulation input parameter vector
5. Upload input data files to remotes
6. Process model files on remotes
7. Run simulation and post-process results on remotes
8. Download output files into labeled directory to host
9. Post-simulation processing, and repeat from 4 for all parameter vectors

Stage 1 includes the starting of a log, preliminary notifications, and testing of machine communications. Stage 2 uploads the files that form the remote-based simulator (including MATLAB m-files or Python files which may make calls to standard simulators such as MCell, or may form a simulator on their own), configures compiles code as required. Stage 3 uploads the simulation model files (if any) to the remote machine. In Stage 4 the user draws a parameter vector to use in a simulation from the set of vectors to be used. The parameters in the vector determine whether some of the model files have to be modified and which data files might be required for a particular simulation (Stages 5 and 6). Once all modeling files are processed then the simulation runs (Stage 7). This might involve generating a script or job file in the host machine, moving it to the remote machine and then invoking a job submission queuing system such as those found in HPC clusters. After each simulation the resulting output data is downloaded (Stage 8). This is trivial in a single-simulation process, but because using multiple heterogeneous computational resources raises the possibility of submitting multiple jobs concurrently, the software needs to ensure the output data is associated with its input vector. Finally, it is necessary to update reports, clean and ready the work directories in the remote machine for the next simulation (Stage 9).

**Figure 1:**
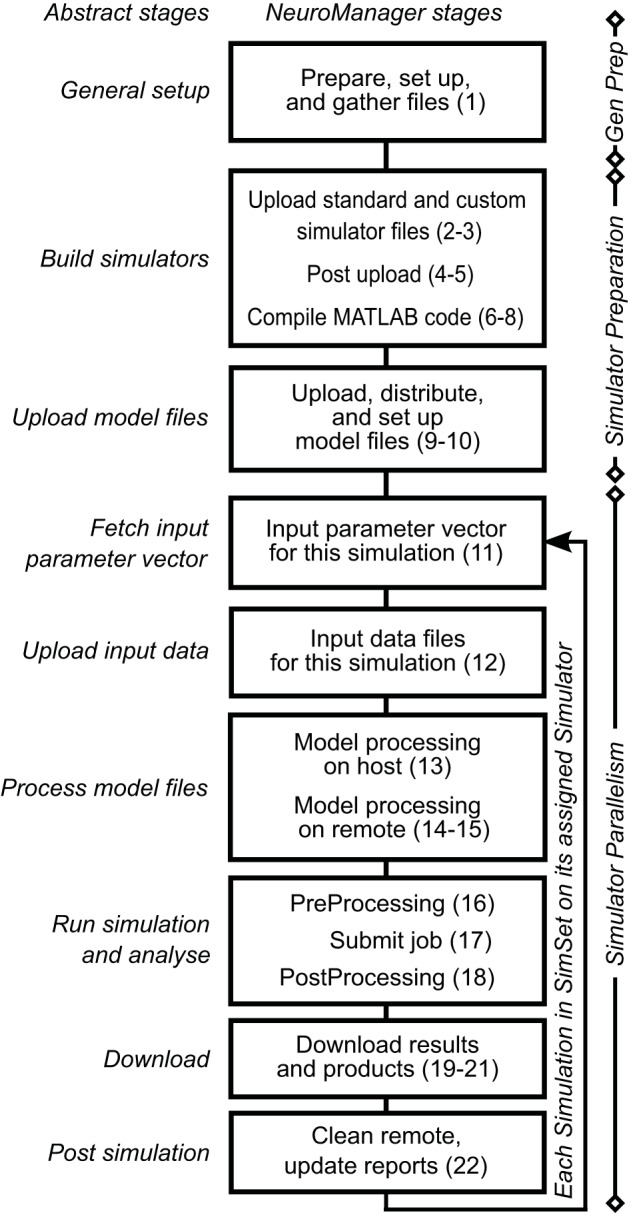
**NeuroManager workflow**. Nine abstract stages (left) are expanded to 22 NeuroManager processes (right). Indications on the right show the location or nature of the process. General preparation (Gen Prep) is done primarily by the host machine; Simulator Preparation, in general, takes place on remote machines; and Simulator Parallelism takes place in all the remote machines at the same time. See Supplementary Material for detailed descriptions of the stages. The numbers in parenthesis indicate the 22 processes.

We expanded these nine workflow stages into a 22 stage Simulation Submission Workflow, Figure 1. We used object-oriented programming to handle intra-stage differences between machines, operating systems, and simulator requirements. Each stage of this workflow corresponds to a NeuroManager class method. A full description of the data structures developed to implement this workflow is in the Supplementary Materials.

## NeuroManager design

### Overall object interaction and flow

**Figure 2:**
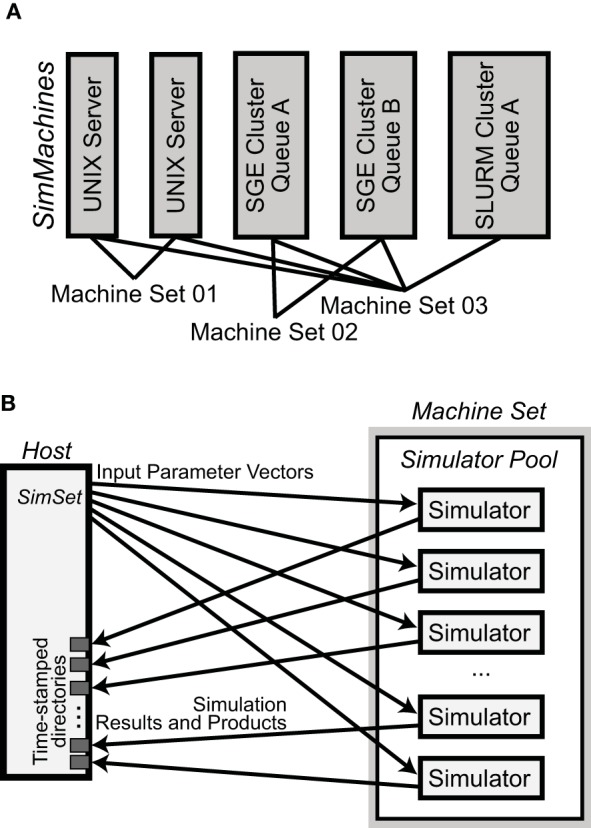
**Basic NeuroManager objects**. **(A)** SimMachines are objects that wrap a computational resource. A Machine Set is a combination of SimMachines. **(B)** A Simulator Pool is the set of all Simulators that have been constructed on any SimMachine in a Machine Set. For each Simulation in the SimSet, NeuroManager gives an Input Parameter Vector for execution to an available Simulator from the Pool. When each Simulation is finished its Results and Products are moved to isolated time—stamped directories in the host.

NeuroManager is run from a **host** computer to perform simulations on **remote** resources. In the simplest case, the host and remote computers are the same machine. Since NeuroManager is an object itself, it can be embedded within larger MATLAB programs as a simulation submission engine, or run from a script. NeuroManager builds objects called SimMachines, by wrapping real machines in classes, then bringing multiple SimMachines together to form a single Machine Set (Figure 2A). Similarly, NeuroManager builds objects called Simulators by wrapping real simulators, e.g., MCell, in a combination of class definitions. The most specific simulator classes are called SimTypes. Each SimMachine hosts multiple Simulators that form a Simulator Pool (Figure 2B). Each Simulation run on a Simulator differs from others by its Input Parameter Vector. The Input Parameter Vector is a set of strings that correspond to parameters used by the simulation. The set of simulations defined by all the Input Parameter Vectors is called a SimSet. The input parameters can be generated by code or stored in a text file called a SimSet Specification.

The Simulator object is composed of a SimCore, a default model, and pre- and post- processing activities. This object contains the static parameters that never change in the development of a sub-project, such as the time step, temperature, cell morphologies, or use of stochastic vs. deterministic algorithms. The Simulator object makes use of a SimCore which is the simulation engine to be used. This could be NEURON, MCell, or a custom executable.

Once the SimSet is built, NeuroManager fetches each Simulation from the SimSet and hands it to an available Simulator from the Simulator Pool using a first-come first-served algorithm. Load-balancing is performed manually at setup by the user's choice of number of Simulators per SimMachine. After a Simulation is complete the Simulator downloads the results into a directory structure on the host machine and tells NeuroManager it is free for another Simulation. This process is the same for each Simulator in the Simulator Pool, thus providing concurrent execution of multiple Simulations across the Machine Set.

### Class hierarchies

**Figure 3:**
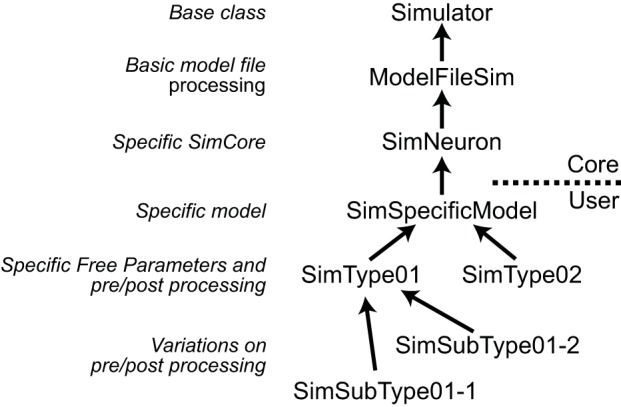
**Simulators are defined hierarchically**. **Left**, the general class hierarchy to implement a Simulator. **Right**, example to implement three different Simulator types. Arrows point to the super-class. The implementation of a Simulator can be divided into Core, provided in NeuroManager, and User-defined sections. The core starts with a Simulator base class, then adds a ModelFileSim subclass which allows to work with files; then a specific SimCore class adds working properties to process specific models, in this case is SimNeuron that allows to run NEURON simulations. Users can then add other functionality specific to a particular simulation with the SimSpecificModel sub-class. Finally, setting up different global parameters to define sub-projects results in individual SimTypes. Further variations produce branching of simulator types.

NeuroManager implements the Simulator, Simulation, SimSet, and SimMachine concepts as distinct software objects. The Simulator and SimMachine objects each have class hierarchies which are diagrammed fully in the User Guide. The *Simulator class hierarchy* uses the Simulator base class to handle most aspects of NeuroManager Simulator operation, with sub-classes to make the Simulator specific to a given SimCore and further sub-classes to make the Simulator specific to a user's Model and research goals. An advantage of this approach is that, as research demands guide the researcher through various simulator/simulation configurations, the nuances are captured in the Simulator object tree, so the researcher can make use of inheritance to simplify the development of a new configuration; see Figure 3.

**Figure 4:**
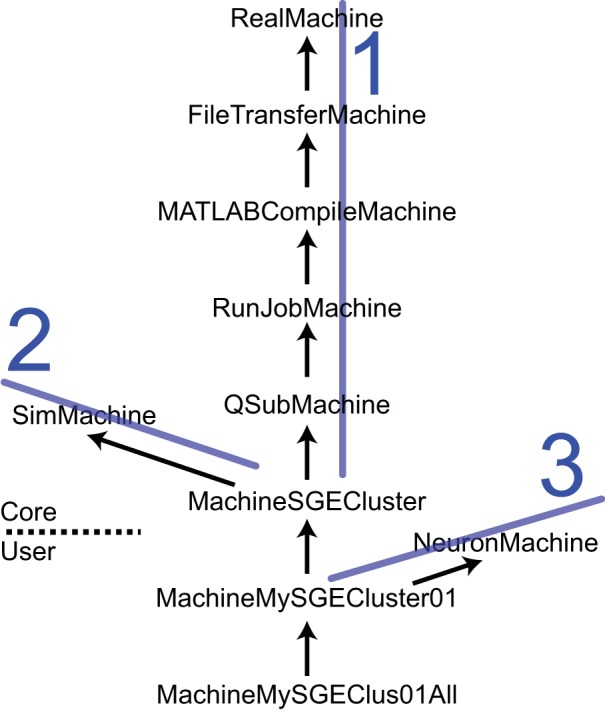
**The SimMachine Class Hierarchy**. A SimMachine is the combination of three inheritance lines of objects. In this example a first ancestral line (1) is composed of classes that provide basic communications; file transfers between host and remote; ability to do MATLAB compilations of Simulator files; basic job submission facilities; and job submission specific to a SGE Cluster. The second line (2) supplies knowledge of how to build and host Simulators. Line 1 and 2 produce a machine class that can host and run Simulators on an SGE cluster. Adding the third line (3) provides location information for the NEURON software installed on a specific cluster. Together they give rise to a class *MachineMySGECluster01* which corresponds to an actual computational resource. Further modifications result in an object that executes in a specific queue in a HPC resource. Classes above the dashed line are supplied with the Core code and those below are user-supplied. Arrows point to the superclass. Full diagram tree in User Guide.

The *SimMachine* class hierarchy provides isolation and inheritance of each element of the heterogeneity of machines and job submission utilities that comprise the Machine Set. An excerpt from the *SimMachine* Class Hierarchy can be seen in Figure 4; the full tree is in the User Guide. There are three ancestral lines which combine to build a SimMachine with full and specific functionality. The first ancestral line provides the infrastructure to run jobs in a specific resource, in this case an SGE cluster. This line is divided in two sections, the first section deals with building a generic machine, using the *RealMachine* class, and then adds the functionality to transfer files between host and remotes, and the configurations necessary to compile MATLAB code. The second part of this line adds the basic and specific job submission procedures culminating in an object that can submit jobs to a SGE cluster (MachineSGECluster). The MATLAB compilation capability, through the *MATLABCompileMachine* class, is a subclass of *FileTransferMachine* because MATLAB compilation requires the ability to transfer files from the host to the remote. The second ancestral line is the *SimMachine* line which provides all the aspects to deal with Simulators and interactions with Simulations. The third is the *NeuronMachine* line that provides location about the simulation engine to be used on the specific machine, in this case NEURON. The three lines are combined using multiple inheritance to produce machine classes that can host NEURON-based Simulators on a specific SGE cluster. Instantiations of this class are Machines used by NeuroManager.

## NeuroManager properties

### Provenance

Provenance is the documentation of the processes and data that have produced a digital object (Simmhan et al., 2005; Moreau et al., 2011). For simulations provenance requires recording the data, processes, and conditions under which a given simulation's output products were obtained, together with those products, with the intent of proof of correctness and reproducibility and test cases and conditions software validation (Miles et al., 2008; Gewaltig and Cannon, 2014). In order to record provenance of neuroscience simulations, NeuroManager puts all program output and simulation results into a time/date-stamped directory; keeps a detailed log of all activities, including machine data and job submission; keeps a copy of input parameter vector files and dynamically constructed or modified model files; records all related software versions; records the versions of the NeuroManager, software, core simulator and user simulator classes; permits the user to add new simulator versions into the simulator class tree; provides utilities to retain other simulation byproducts as desired; and records most scripts and software output and error output.

### Interaction with clusters and HPC resources

NeuroManager interacts with cluster submission managers, such as SGE and SLURM using SSH2. Queue identity, number of cores/nodes, timeouts, and other job characteristics are specified through the job file which the user controls either directly, through sub-class settings, or through the Input Parameter Vector. NeuroManager writes the job file for the user and automates choice of names, file locations, and compilation location, and provides user notification. With NeuroManager the user interacts with the host machine and the software takes care of all the uploading, compiling, job submission, collecting results, and downloading to host automatically.

### Monitoring

NeuroManager allows the user to monitor simulation progress in four ways: (1) Logging; as described above; (2) Webpage; the MATLAB interface provides a built-in browser which we use to present the current state of all simulations; (3) Remote operation; through the use of the UNIX *screen* utility; (4) User notifications; NeuroManager provides SMS and/or email messages for essential points in session evolution which can be programmed globally or on a simulation-by-simulation basis and could include attachments such as plots.

### Scheduling and resource allocation

NeuroManager's Simulation scheduling algorithm is a First Come First Served approach (Kacprzyk et al., 2008). Once all of the Simulators in the Machine Set have been constructed NeuroManager begins placing Simulations on Simulators. Starting at the beginning of the Simulator Pool the first available Simulator will be given the next Simulation in the batch. The Simulations are all presumed to be equal in communications requirements and computational load (footprint and number of machine operations), which is appropriate except in the situation where the user is specifically changing number of simulation steps in the input parameter vector. The Simulations are also considered independent and of equal priority and are scheduled in the order in which they appear in the SimSet, which has no specified completion time or performance criteria. In our current approach, the Simulators are assumed to be independent, identical, and static—that is, they are not affected by each other, have equal computational power and resources, and are not affected by external workload.

## Requirements and performance

### Installation and requirements

A detailed installation and configuration procedure is described in the User's Guide. Briefly, NeuroManager requires:

- A MATLAB version 2013a+ with compiler toolbox. In the simplest installation the host machine will have the compiler. However, it is possible to have a remote compiler machine that NeuroManager can use to produce executables. This allows MATLAB student versions to be used as a host. As part of the configuration of NeuroManager all necessary files are gathered, moved to the compilation machine and then moved to the remote machines. All this is done automatically. Thus, it is possible to run NeuroManager with a single compile license.
- Host machines can be Windows (tested on 7, 8, and 10) and UNIX (tested on Centos 5.6 and 6.6). Remotes have to be UNIX machines. Windows machines have to have freely available Putty (Tatham et al., 2006) (included in NeuroManager). All remotes have to have SSH2.
- A compatible Java version.
- The MATLAB ssh2-v2 library (Freedman, 2015) (included in NeuroManager).
- Some XML examples make use of the free HE edition of the Saxon processor from Saxonica installed on the host (Saxonica, 2015).
- The SimCore (simulator engine) has to be available in each remote machine.
- All remote machines have to have the appropriate freely available MATLAB MCR (Mathworks, 2015c).

NeuroManager can run from the MATLAB graphical interface, but also runs without the use of the desktop, which for UNIX hosts permits remote operation. The User Guide gives details on how to operate NeuroManager remotely. All software is available at https://github.com/SantamariaLab/NeuroManager as well as per request to the authors.

### Configuration

Configuration of NeuroManager involves defining the machines to be used for simulations, the Simulators to be used, and the parameters used for individual simulations. Each of these tasks is done using object-oriented classes in individual MATLAB m-files. We provide examples to assist the user with the configuration procedure. Some of the examples make use of Python on the remote machine. The code is published with an open source license.

We have tested NeuroManager, both independently and simultaneously, with eight machines in many configurations: two multi-core UNIX servers, four queues on a local UTSA cluster (CBI, 2015), and three queues on the Stampede cluster at the Texas Advanced Computing Center (TACC, 2014). We have run sets of simulations with runtimes ranging from a few seconds to several days.

### Tests

We tested NeuroManager on two multi-core servers and two queues in a cluster (CBI, 2015). The tests consisted of running a simulation of a neuron using the NEURON simulator. Stand-alone, this simulation running on our reference server, took between 5 and 30 min depending on the parameters of the particular test being studied.

We defined the temporal overhead as the total session time minus the SimSet time. The SimSet time consists of fetching an Input Parameter Vector and running the actual simulation plus moving the necessary input/output files for all the simulations. We calculated the overhead of NeuroManager as a function of the number of simulators on single machines and clusters. For a session in the reference server consisting of 12 simulations on two concurrent Simulators the run time was 3950 s with a SimSet time of 3741 s which results in an overhead of 209 s. As the number of Simulators increased to 12 the session time dropped to 1183 s with a SimSet time of 959 s and an overhead of 226 s. We also calculated the overhead of SimSet for the 12 Simulator example. The average execution time of all simulations was 780 s, thus the total SimSet overhead was 179 s for 12 simulators which results in an average overhead per simulation of 15 s. We repeated this same procedure in a queue of the cluster. For the case of 12 simulators, distributed over 12 nodes, the run time was 1408 s, the SimSet time was 1242 s resulting in an overhead of 166 s. The SimSet overhead was 825 resulting in an average overhead per Simulator of 35 s. We repeated this for different numbers of Simulators, from 2 to 12, in the server and the cluster. In all cases the overhead remains practically constant for each particular machine. Consequently, the overhead fraction of the total session time decays as the number of Simulators increases. The overhead also remained constant when the number of simulations performed increased.

We determined the performance of NeuroManager by distributing a fixed number of Simulators over 4 configurations of SimMachines mixing servers with clusters. The session time for 2 simulators on Server 1, 2 on Server 2, 8 on Cluster 1 and 8 on Cluster 2 was 1536 s. For another simulation with 8 simulators on each server and 2 on each cluster was 1566 s. For all simulators on the servers the session time was 1326 s and for all simulators on the clusters it was 1488 s. Thus, we obtained similar performance independent of the distribution of Simulators. Finally, we tested NeuroManager's stability, the property of obtaining identical behavior for identical tasks, by running a 17 min session on four SimMachines with 12 Simulators 10 times. The average session time was 1028 ± 19 s (STD) and the average SimSet time was 713 ± 13 s.

## Using NeuroManager

Here we provide an overall description of the main programming and conceptual properties of NeuroManager starting with the execution of MATLAB code in one machine. Explanations with more detail can be found in the User Guide and accompanying example programs.

### Basic simulation

**Figure 5:**
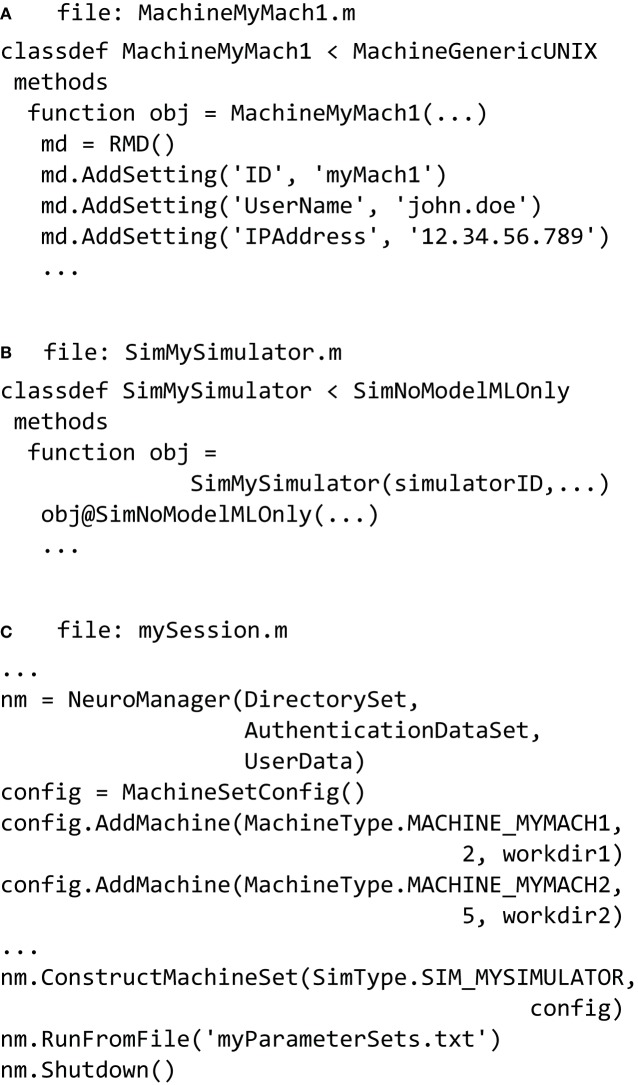
**NeuroManager setup**. **(A)** Define at least one SimMachine. This example defines *MachineMyMach1* as a sub-class of *MachineGenericUNIX*, with corresponding machine type of MACHINE_MYMACH1. **(B)** Define a Simulator class that specifies a particular Simulator/Free Parameters setup. In this example, it is a simulator called *SimMySimulator* that is MATLAB only. This class has a corresponding SimType of SIM_MYSIMULATOR. **(C)** Write a session script that constructs NeuroManager. Use the SimMachines defined in A and specify the number of Simulators and work directory for each SimMachine, construct the MachineSet with a specific SimType SIM_MYSIMULATOR defined in **(B)**. Finally, run the simulations defined by the Input Parameter Vectors in **“**myParameterSets.txt.”

In the simplest case the host machine is the same as the remote machine. The definition of a new machine is done in a file called something like **MachineMyMach1.m** (Figure 5A) and stored in a subdirectory called **LocalMachines**. In that file we define an individual machine as a new class based on a superclass. We provide super-classes for a generic UNIX machine, an SGE Cluster, and a SLURM cluster. Referring to Figure 5A, we describe the fundamental properties of a machine class file. The machine is defined as a class definition using

(1) $$\begin{array}{l} classdef\;MachineMyMach1<MachineGenericUNIX \end{array}$$

which declares that the new object will be a subclass of the *MachineGenericUNIX* class and will inherit its properties and methods. The class constructor

(2) $$\begin{array}{l} function\;obj=MachineMyMach1\left(\ldots \right) \end{array}$$

will create an instance of that class; its inputs are structures or variables that define the machine ID, local directories and other information necessary set up the machine properly.

Simulators, also defined using a class hierarchy, typically form a wrapper around a common simulator such as NEURON or MCell, but can also be built using MATLAB code, Python, or other languages. Simulators are formed from a pair of m-files: the class file that operates on the host, and a file called **userSimulation.m** that operates on the remote. We provide MATLAB-only, NEURON, and MCell classes, and examples of their partner **userSimulation.m** files. Each class has functions and properties particular to the simulator to be used. Once the user defines a simulator then it has to be added to the *SimType* class that defines all the simulators available to the user. In the example seen in (Figure 5B) the *SimMySimulator* class is a subclass of the *SimNoModelMLOnly* class, which is for simulators that are composed of MATLAB code only and have no model files to process.

Finally a session script file called, for example, **mySession.m**, brings together the machines, the simulators, and the parameters to be explored (Figure 5C). First we construct an instance of the *NeuroManager* class with location of simulation files, SSH authentication key information, and user information for text or email notifications. The SSH key information allows NeuroManager to log in and work on remote machines without the need for user intervention once the private key passphrase has been entered. The object generated by the *NeuroManager* constructor (here called “nm”) provides methods that direct all the functions of NeuroManager. Next, we tell NeuroManager which of our predefined machines to use for this session by constructing a *MachineSetConfig* object and adding machines using its addMachine() method. The resulting machine set could be a single server or a collection of heterogeneous machines, e.g., local servers and clusters, defining a “Machine Set” (Figure 5). Each call to addMachine() requires the MachineType as described in Figure 5A, the number of virtual simulators to run on each machine in parallel, and a user—provided remote work directory. The set of all simulators forms a simulator pool that NeuroManager will use to run simulations. For example, machine MY_MACH1 can be added with two simulators and machine MY_MACH2 can be added with five simulators, forming a pool of seven simulators. When the session script runs with a set of 20 simulations, then NeuroManager will place two simulations on MY_MACH1 and five on MY_MACH2. As each simulation finishes and its simulator becomes free, another simulation is placed on it until all the parameters sets have been run.

In order to run the simulations (Figure 5C) we use the *NeuroManager* class's runFromFile() method. Each line of **myParameterSets.txt** defines a vector of input parameters that will be inputs to individual simulations via the userSimulation() call running on the remote. After all the simulations in **myParameterSets.txt** have been completed, we call the *NeuroManager* class's shutdown() method to clean scratch directories, create reports, and do other housekeeping tasks.

We provide examples of using a local UNIX server in combination with a local cluster and an HPC resource. NeuroManager handles the interactions from the host to each of the machines, including job submission protocols and retrieval of simulation output files. We also provide examples of using different simulators. One of our examples is a trivial simulator called “SineSim” which is entirely in MATLAB and plots sine waves based on two input free parameters: frequency and duration. A class called *SimSineSim* (a subclass of *SimNoModelMLOnly*) and its companion remote-side function userSimulation() work together to form the sine wave and plot it.

### Using NeuroManager with neuron

**Figure 6:**
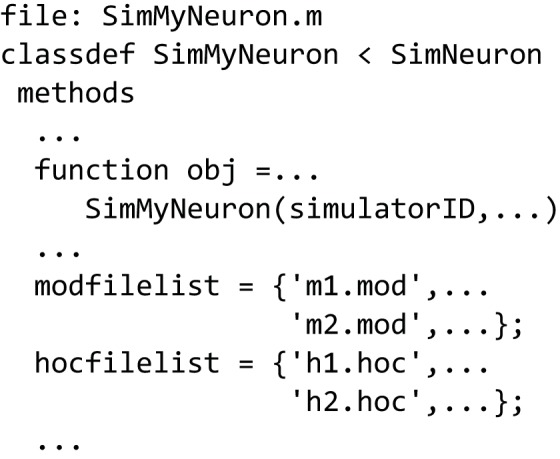
**Defining a NEURON-based Simulator class/type**. Sub-class *SimMyNeuron* is based on class *SimNeuron*, which itself adds NEURON-specific functionality to the *ModelFileSim* class. The list of mod and hoc files that define the model can be hard coded in this file. The class provides functions to modify or construct mod and hoc files before or during execution. The user can also do custom pre- and post-processing in this new class based on *SimNeuron*.

Similar to defining a simulator to run MATLAB-only code (Figure 5B), we can use the *SimNeuron* class to build a virtual simulator based on the NEURON simulator (Figure 6). NEURON simulations manage hoc and mod extension files which can be named in the new subclass or dynamically constructed or modified using class methods. The hoc files are used to define the structure of neurons and general simulation processes, while the mod files are used to describe biophysical mechanisms used by the simulation. We provide examples that incrementally describe how to use the different parts of this class.

### Varying ion channel distribution in a NEURON model using hoc and python languages

Here we use NeuroManager to investigate the effect of varying the concentration of an ion channel in a Purkinje cell model (Hines et al., 2004), ModelDB Model 17664 (Miyasho et al., 2001; ModelDB, 2015). We have modified the available hoc and mod files for compatibility with NeuroManager (primarily by removing all GUI elements) and we have made use of Python to define the simulation input current placement and characteristics and the place where voltage data will be collected. NeuroManager runs the simulations with the given parameter sets, plots the results, labels the plots, and ships them back to the host together with time and voltage data.

We form a subclass of the *SimNeuron* class called the *SimPc* simulator class, which inherits all the *SimNeuron* facilities. In addition, it deals with both the list of mod files which determine the behavior of the biophysical mechanisms used, and the **Purkinje.hoc** file which holds (1) cell morphology information, including soma, smooth dendrites, and spiny dendrites; (2) global electrical characteristics such as axial resistance; and (3) distribution and characteristics of each biophysical mechanism to be inserted into each model section. Then we create a subclass of *SimPc* called *SimPcKh* which adds the ability to distribute the anomalous rectifier channel, Kh, differentially to the three section types (soma, smooth dendrites, and spiny dendrites) on a simulation-by-simulation basis. To achieve this, during the *PreRun Model Processing Host* workflow stage for each simulation we assemble a new **Purkinje.hoc** containing the simulation-specific Kh insertions as well as the morphology and static biomechanisms. This approach puts as much simulator—specific processing directly in the *SimPcKh* class, so that the uploaded m-files are as simple as possible and the modification process is made an integral part of the class. Should we change our process, we can make a new subclass called *SimPcKh02*, then both versions of the simulator are inspectable and available for use in the future. In this way, we have a clear, usable track of the trajectory of simulator evolution. In addition, every simulator class has access to the session log, so that users can make log entries during simulator construction, hoc file modification, or whenever a simulator method is employed. When all is ready on the remote, each simulation will run with its simulation-specific Kh concentration values for soma, smooth dendrite, and spiny dendrite segments. The actual NEURON simulation on the remote is done as a Python program importing the NEURON module.

### Varying ion channel characteristics in a NEURON model specified using NeuroML

SimCores such as NEURON can handle models specified in the NeuroML format (Gleeson et al., 2010), while others cannot. In the first case, NeuroManager's approach is to edit the NeuroML model file using XML techniques in response to a Simulation's Input Parameter Vector, then send the resulting XML file to the SimCore as is. If the SimCore cannot understand NeuroML, NeuroManager's approach is more involved: to edit the NeuroML model file using XML techniques, then convert the edited XML file into an input the SimCore can understand.

Although NEURON can handle NeuroML input, we present the latter, more illustrative case—automatically turning a NeuroML-format model file into standard NEURON mod file format before its compilation into the mod library and subsequent use in the simulation. We developed an example called “NeuroML01,” which is a soma with two types of ion channels, NaF and Khh, and a Leak channel. We use Padraig Gleeson's NeuroML specification of the NaF channel from the NeuroML website (Gleeson, 2015b), and pull eight parameters from it as Free Parameters to be specified in the Input Parameter Vectors (mAlphaA, mAlphak, mBetaA, mBetak, hAlphaA, hAlphak, hBetaA, and hBetak). The corresponding Simulator class (*SimNeuronSimpleSpike03*) modifies the XML channel specification according to the Input Parameter Vector for each simulation, transforms the modified XML file into a NEURON mod file using Gleeson's XML stylesheet supplied at NeuroML's Source Forge presence (Gleeson, 2015a), and uploads the new mod file to the remote for compilation and simulation. In this example, we run nine simulations with different Input Parameter Vectors, each corresponding to a different configuration of the NaF ion channel. All other parameters are static and set within the Simulator. The NeuroML01 example is presented as a working session in the User Guide.

### XML NeuroManager sessions

We developed an XML format for single NeuroManager sessions that consist of single SimSet multiple Simulations on a configured Machine Set. Called NMSessionML, the format is host-OS-independent and language-independent. NMSessionML may be useful in integrating NeuroManager with other systems. We have provided three examples (NMSessionML01-03) that do the same simulations as examples SineSim03, SimpleSpike02A, and KhStudy, but run from NMSessionML files instead (see the User Guide for details).

**Figure 7:**
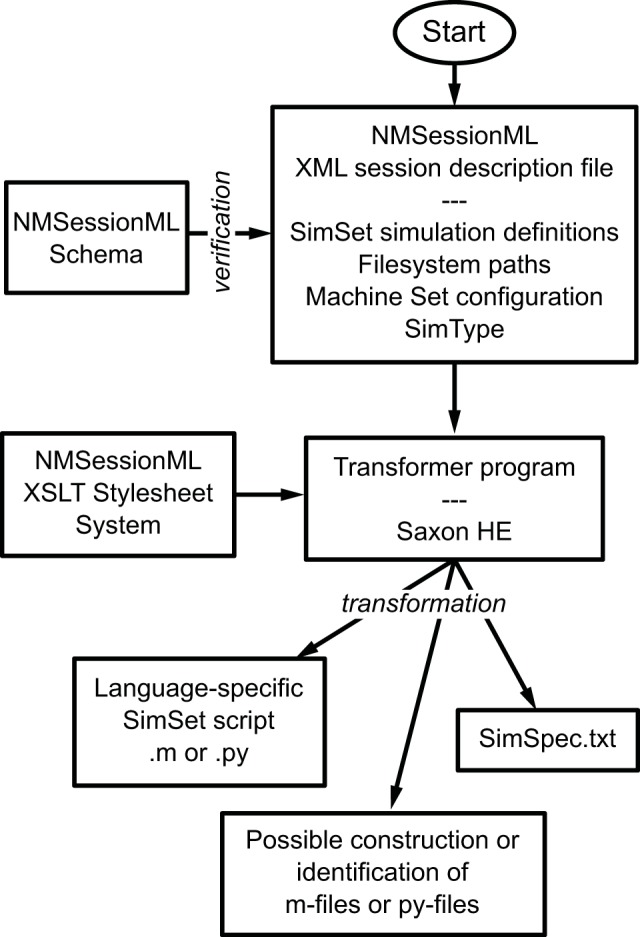
**Using NMSessioML to run a set of simulations**. The user describes the Session in a single XML file, specifying the SimSet, MachineSet configuration, and SimType, then NeuroManager makes use of an XSLT stylesheet system and a transformer program such as SaxonHE to write the Session and SimSpec files and finally run the simulation.

The basic flow of NMSessionML action can be seen in Figure 7. The user creates the XML file, which specifies the SimSet, the Simulator type, the individual machines composing the Machine Set, user information, and file locations. The run script uses the NMSessionML Schema to check the XML file's format, then employs custom stylesheets to produce the SimSpec data file and the language-specific script file required to run the session.

## Discussion

In this work we described NeuroManager, an object-oriented tool that simplifies computational neuroscience workflows in heterogeneous computing resources and multiple simulator types. Presently, the majority of the efforts in computational neuroscience are centered in either developing simulation environments or providing easy access to HPC resources. Our software provides the infrastructure to leverage computational resources, manage the use and evolution of simulators and organize the potentially large amounts of data generated. As such, NeuroManager provides a tool that will allow users to manage, analyze, and improve their simulations in an orderly environment.

### Workflow and software design

Workflow modeling and management are a vital part of current and future neuroscience as the era of Big Data continues to evolve and scientific progress is increasingly multidisciplinary and collaborative (Chen and Zhang, 2014). Designing NeuroManager with an object-oriented approach allows flexible workflow management. The design of NeuroManager is to relate a workflow stage to specific processes related to simulation submission. As a consequence, the workflow stages are not independent modules. Other approaches to workflow automation generate modules that can be separated and distributed over different resources (Korkhov et al., 2007; Wibisono et al., 2007; Yu et al., 2008; Cushing et al., 2014). For example the WS-VLAM (Web Service-Virtual Laboratory Amsterdam) project approach is to make each element of a workflow an independently-running component, then to farm those components out to remote computational resources (Korkhov et al., 2007). Workflow components communicate by making use of channel services constructed for that specific purpose. While highly flexible and scalable this approach requires full grid-level support. In contrast, NeuroManager's approach is to use a fixed workflow and keep the stages internally flexible. NeuroManager tailors workflow stages to specific actions, most of which do not require HPC resources. Moreover, NeuroManager does not require any sort of grid-level installation, and can make use of any clusters or servers to which the user has access. As a result, NeuroManager is more readily available to the individual scientist.

The design of NeuroManager might be construed as a MapReduce framework (Sakr et al., 2013; Radenski, 2014). NeuroManager has a Map() stage in which the Simulations to be performed are distributed on the SimMachines. After the simulations are done, the Reduce() process is to run automated analysis and gather the outcomes, e.g., plots or summary files. We have used the terms “host” and “remote” rather than “master” and “slave” because NeuroManager does not have direct control over access to the remote nodes. As such, other tasks or users could be sharing the same resources. Overall, NeuroManager operates as middleware to virtualize hardware, software, and users.

### Limitations

As is the case with all software tools, there are limitations to their use. In the case of NeuroManager the limitations are related to the use of commercial software, parallelization, and overhead. We designed NeuroManager to minimize the use of licenses. The advantage of using MATLAB is access to validated software whose support does not depend on grant funding. This allows NeuroManager to have a longer horizon of usability. Nevertheless, we are currently developing equivalent Python code.

NeuroManager is not an HPC scheduler; instead it makes use of job submission utilities to run simulations on large resources. Our software is focused on providing the infrastructure to track simulations and their evolution. Potentially, NeuroManager could integrate more sophisticated scheduling and load leveling algorithms. The object-oriented approach provides a flexible environment for such modifications.

The overhead of NeuroManager is variable; however, the largest bottleneck is the MATLAB compilation. In our experience this can take from 90 to 180 s depending on machines used. So, taking into account this it would seem that NeuroManager is best used for simulations that require many minutes to days to run. Very short simulations will likely incur too much overhead to be efficient time-wise; however, the time and effort savings in user workflow automation such as file transfer, figure production and labeling, may justify even such use. If a user has 10,000 short simulations to run, they could be combined into a 100 × 100 approach that would minimize overhead and maximize the effectiveness of using HPC resources. Meanwhile, the user would gain all the workflow advantages of having plots automatically labeled correctly and not having to upload or download manually, all of which could take a great deal of time without the use of NeuroManager.

When using heterogeneous resources the first-come-first-base scheduling algorithm could face bottlenecks if the performance of the machines differs widely because the software assumes that all Simulators are equal. This could be overcome by adding performance description to the SimMachine for particular Simulators, thus allowing NeuroManager to better schedule jobs.

### Related work

There is increasing interest in the development of tools to annotate, track, and organize the simulation or analysis of data in areas of engineering and science. The tools most related to computational neuroscience are Sumatra, Lancet, and Mozaik. Sumatra (Davison, 2012) is a simulation project manager designed to demonstrate replicability via close integration with a version control tool. The user “checks out” the manager and model files, then runs the simulations, which are automatically placed into a database, under version control. Sumatra automatically scans files for dependencies in order to capture as much metadata as possible, and collects some scientific/experiment context. Although the two approaches are not compatible in their current configurations, the concept that Sumatra investigates is a powerful one and might be useful in future work on NeuroManager.

Lancet (Stevens et al., 2013) is a Python application that combines with IPython Notebook (Perez et al., 2011; Shen, 2014) to allow the researcher to grow a scientific workflow that involves neural simulations. When in daily use, Lancet allows the researcher's workflow to evolve naturally, and keeps track of its changes. Lancet creates a scripting language for its operations that can be intermingled with notes via IPython Notebook. In contrast, NeuroManager uses a fixed workflow that is adapted by the user to research needs through object methods for each stage. This more formalized, fixed workflow approach simplifies the use of available computational resources, makes multiple parallel simulations more observable, allows instant access to all simulator versions, and facilitates sharing between groups. Like NeuroManager, Lancet has a “simulator” object which can handle a remote simulator, but it does not allow the definition of a Machine Set. Lancet uses the simulator object primarily to make the software simulator-independent. Although this is true of NeuroManager, it also uses the simulator object to be able to host multiple Simulators on a Machine Set, to customize pre- and post-simulation scripting, and to allow a tree approach to simulator evolution.

Finally, Mozaik is a resource focused on managing simulations of 2D neural networks (Antolík and Davison, 2013a,b), which uses PyNN (Davison et al., 2008) for simulator type independence, Neo for data structuring (Garcia et al., 2014), and Matplotlib for data plotting (Matplotlib, 2015). The software is modular, enhancing extensibility and customizability. The fundamental design of Mozaik is specific for 2D neural networks. NeuroManager, in contrast, is not specific to a given simulation type.

The automation of complex workflow is becoming more common in biological sciences. For example, Taverna (Oinn et al., 2004) is a tool for working with web-based bioinformatics databases, and Vistrails (Callahan et al., 2006) has the goal to move visualization processing pipelines beyond the tedious single-configuration-by-click approach by facilitating the production of many visualization products at one time.

### Interaction with other computational neuroscience resources

NeuroManager has been tested with NEURON, MCell, and MATLAB-only simulators, but its workflow-based approach should be suitable for integration with MOOSE (Bhalla et al., 2011), PyNN, and the Neo object model. Neuromanager could also be extended to accept models built using neuroConstruct (Gleeson et al., 2007), a tool that allows users to construct 3D neural models in the NeuroML format.

NeuroManager could interface in the future with other resources and tools. The Neuroscience Gateway is a large-scale facility that acts as a portal between the neuroscientist and various neuroscience simulators hosted on HPC resources (Sivagnanam et al., 2013). While the Neuroscience Gateway takes charge of many aspects of simulations management, NeuroManager, once configured, provides the flexibility and independence of using both local and HPC resources and is capable of acting within other software as an embedded simulation engine.

There are efforts to standardize the representation and reporting of simulations in systems biology. One important effort is around the development of SED-ML, which is an evolving standard for simulation provenance (Köhn and Le Novere, 2008; Waltemath et al., 2011). Although SED-ML deals with the use of “simulation procedures” and “simulation algorithm,” “model,” and most other aspects of daily simulation work, they do not implement the concepts of “simulator” or “machine.” In order to better to interact with NeuroManager it would be necessary to implement the concepts of Simulator and Machine within SED-ML.

NeuroManager could make use of the Python-based package called RADICAL-SAGA (RADICAL, 2015), which is a light implementation of the SAGA access layer to heterogeneous distributed computational resources (Merzky et al., 2015). Although there is no MATLAB binding of SAGA the Python binding has adaptors for the two cluster types mentioned here (SGE and SLURM).

Although outside the scope of this paper, NeuroManager's workflow automation should prove useful for parameter space search and optimization. The *NeuroManager* class's runFromSimSpec() and runFromFile() methods return a results structure that contains each simulation's SimID, the location of its results, and its success or failure. A script embedding NeuroManager can access this structure, process the results, and create and run a new SimSet based on its search algorithm. NeuroManager's use of a unique SimID for each Simulation enables both single-threaded and multi-threaded search algorithms to operate simultaneously within a given SimSet. In this form, NeuroManager could interact with Neurofitter (Van Geit et al., 2007), an application designed to investigate parameter spaces in neuroscience. Other important improvements will be to incorporate MATLAB's Global Optimization Toolbox (Mathworks, 2015b) and the *scipy.optimize* Python module (SciPy.org, 2015). As we have mentioned before, the object-oriented properties of NeuroManager give it the flexibility to incorporate these tools.

### Future work

In future versions of NeuroManager, we expect to provide checkpoint management, high-level fault tolerance, handling of assigned timeouts, and a more sophisticated scheduler. Another useful addition to NeuroManager would be the ability to add and/or subtract individual machines, simulators, and simulations while NeuroManager is running. Also beneficial will be integration with database facilities for cataloging inputs, configurations, comments, results, and analyses. MATLAB has facilities for interaction with databases, such as the Database Toolbox (Mathworks, 2015a). Other efforts in this area (Günay et al., 2009) may be compatible with NeuroManager's construction. We also hope to add provenance facilities that are more formal and that allow submissions to ModelDB that not only include model and hoc files, but also simulator class files, specification files, and other related files that are sufficient to recreate simulations in full.

We are aware of NeuroManager's potential role in integrating experiments involving both electrophysiology and computational neuroscience simulations. This will require the development of object classes that deal with the analysis of experimental results which could then interact with modeling results.

## Funding

NSF-EF 1137897, NSF-DBI 1451032, NIH-G12MD007591 (for use of computational facilities at UTSA), Texas Advanced Computing Center for providing HPC resources.

### Conflict of interest statement

The authors declare that the research was conducted in the absence of any commercial or financial relationships that could be construed as a potential conflict of interest.
