# Supplementary material for: NeuroManager: a workflow analysis based simulation management engine for computational neuroscience
Source: Front Neuroinform. 2015 Oct 13;9:24. doi: 10.3389/fninf.2015.00024 (PMC4602303; doi:10.3389/fninf.2015.00024)
Supplement: Supplementary file 1 [file Presentation1.PDF]

Supplementary Material: NeuroManager: A Workflow Analysis Based Simulation Management Engine for Computational Neuroscience

David B. Stockton and Fidel Santamaria*
*Correspondence:
Fidel Santamaria
fidel.santamaria@utsa.edu

NEUROMANAGER WORKFLOW

The workflow of simulation submission takes place in stages that are common across all simulator and machine types. Using our experience running Matlab, Neuron, and MCell simulations on Linux servers and SGE and SLURM clusters we defined a simulation workflow composed of 22 stages. Since the stages were defined to cover all the different simulations and submission requirements not all stages are applicable to all simulations. Each stage is linked to a method in one or more NeuroManager classes (presented in parentheses). Often a method named after a workflow stage is declared at one class level as an abstract method but is made concrete in subclasses in order to accommodate machine or simulator differences. We list and describe each stage. A flow diagram is shown in Figure 1 in the paper proper.

1. *Pre Upload Files* (`preUploadFiles()`) — Actions that have to take place before actual upload to remote or compile machines takes place. These activities include provenance work such as recording software versions and hardware configurations, as well as actions related to file upload such as fetching files from lab servers.

   This stage is unique since it has machine aspects and simulator aspects. Accordingly, there is an abstract declaration in ***Simulator*** with concrete definitions in the major simulator types (such as ***SimMCell***). There is also a concrete definition in ***SimMachine*** and extensions of that definition in the major machine types (such as ***MachineGenericUNIX***.
2. *Upload Standard Files* (`uploadStdFiles()`) — Upload of NeuroManager core remote–bound m-files to compilation machine and other core remote–bound files to the remote.

   The `uploadStdFiles()` method in ***Simulator*** calls the machine method `uploadStdSimulatorFiles()` in ***MATLABCompileMachine***, since machines know 1) where the source directory is and 2) about cross–compilation — the simulator does not.
3. *Upload Custom Files* (`uploadCustFiles()`) — Upload of the user–supplied **userSimulation.m** file that is NeuroManager's interface to the user's simulations and required for all simulations, to the compile machine. Also includes additional user–supplied simulator files other than **userSimulation.m**, such as supporting m-files, the MCell executable, or Neuron–associated Python files. m-files are uploaded to the compilation machine; all others go to the remote.

   Similar to `uploadStdFiles()`, this method in ***Simulator*** merely asks the machine to upload the files via `uploadCustSimulatorFiles()` in ***MATLABCompileMachine***. Since custom files

may also include non–m–file files, the list is split and the m–files are sent to the compilation machine; the remainder go to the remote (execution) machine.

4. *Post Standard Files Upload* (`postStdFilesUpload()`) — Actions concerning standard uploaded core files that must occur after *Upload Standard Files*, such as making a copy of the machine data file on the remote.
   Defined and used in ***Simulator***.
5. *Post Custom Files Upload* (`postCustomFilesUpload()`) — Actions on any custom files that must occur after upload, for example, making a file executable or copying files to common areas on the remote. For example, we use this stage to log the MCell executable's version, since this is the first opportunity NeuroManager has to run the executable to get its version.
   The abstract declaration is in ***Simulator*** and the concretes are in the major simulator classes such as ***SimNoModelMLOnly*** or ***SimMCell***.
6. *Pre Compile* (`preCompile()`) — Actions relating to MATLAB compilation that must occur in the host before the compilation takes place such as the creation of a script files that will perform the MATLAB compilation.
   Defined in ***MATLABCompileMachine*** and used by ***Simulator***, this method creates the compile command, places it into a shell script file for use by *Compile*, uploads it, and makes it executable.
7. *Compile* (`compile()`) — Performing the MATLAB compilation on the compilation machine by execution of the shell script provided by the *Pre Compile Stage*.
   This operation has some time footprint so we do it in parallel on the remotes; accordingly the shell script creates a signal file indicating completion and completion status. Note that we only compile once per machine, for the first simulator constructed on that remote machine; the remainder of the simulators use copies of or links to the compilation products. Defined in ***MATLABCompileMachine*** and used by ***Simulator***.
8. *Post Compile* (`postCompile()`) — Actions relating to MATLAB compilation that must occur after the compilation takes place, such as copying of relevant compilation products into common areas. This stage is important when the compilation is done in a different machine than the remote; the executables are transferred from the compilation machine to the machine on which the simulation will be run.
   The actual transfer is compilation machine to host to remote machine using a subdirectory of the MachineScratch directory on the host. Defined in ***MATLABCompileMachine*** and used by ***Simulator***.
9. *Upload Model Files* (`uploadModelFiles()`) — Upload of the simulation model files, such as the mdl files of an MCell simulation or the mod and hoc files of a Neuron simulation, to the execution machine.
   Similar to the standard and custom uploads, this method in ***ModelFileSim*** merely asks the machine to upload the files via `uploadModelSimulatorFiles()`, which has definition in ***SimMachine***.
10. *Post Model Files Upload* (`postModelFilesUpload()`) — Actions on any model files that must occur after upload. For example, copying of model files in the remote machine to common areas.
    The method is part of the ***ModelFileSim*** class and uses `remoteCopy()` from the ***FileTransfer-Machine*** class.
11. *Fetch Simulation Parameter Vector* (`fetchSimulationParamStr`) — Retrieving the next input parameter vector for use by the simulation. The vector of input parameters is defined by a line in the SimSet, as described in the User Guide, and is loaded into the Simulation's Parameters property. When the commandline for running that specific simulation is constructed, `fetchSimulationParamStr` pulls the parameters from the simulation object and constructs the required string. On the remote,

that string will be passed to `userSimulation()` in the *varargin* parameter, so the user can access the parameter values. Note that the input parameters are also available on the host through the `getParam()` function, which access the Simulation object itself.

`fetchSimulationParamStr` is defined in the ***Simulator*** class.

12. *Upload Simulation–Specific Data Files* (`uploadInputDataFiles()`) — Upload of data files that the individual simulation will use for input, into the simulation's input directory from the user's custom directory. For example, the user may have input signal files, or tables of ion channel characteristics by segment, or various types of experiment data to use as input. Note that the data files themselves are simulation–specific, but the need to upload it is a characteristic of the simulator. The data file name can be specified in the simulation input parameter set if desired.

    This method is found in the ***Simulation*** class and is one part of a pair with `defineSimulationInputDataFiles()` in the ***Simulator*** class, which needs to be overridden in subclasses to actually choose files; the ***SimSineSim*** class shows this in use.

13. *Pre Run Model Processing Host (H)* (`preRunModelProcPhaseH()`) — Actions dealing with model file processing that originate on the **host** machine before the simulation is run, such as copying model files from simulation common to the simulation's input directory on the remote, creation of a model file compilation shell file (Neuron), creation of a master model file (MCell), or assembly of a modified mod file (Neuron).

    This method has abstract declaration and is actually run in ***Simulator***. In the ***NoModelFileSim*** class its concrete version is merely a no–op since there are no model files to work with. Otherwise, major simulator classes define what they need done on the host regarding model files (such as constructing new simulation–specific ones).

    The ***SimNeuron*** class introduces two abstract methods called from within `preRunModelProcPhaseH()` that allow the user to modify hoc and mod files, called `preRunModelProcPhaseHModFileModification()` and `preRunModelProcPhaseHHocFileModification()`, respectively. Subclasses of ***SimNeuron*** make these concrete. The supplied examples show how to use these.

14. *Pre Run Model Processing PreSubmission (P)* (`preRunModelProcPhaseP()`) — Actions dealing with model file processing that must occur on the **remote** machine before the simulation is run, but that cannot be part of the actual job submission. For example, when submitting a job to the TACC NORMAL queue, which does not allow compilation of Neuron mod files as part of the job, the compilation must done before the job is submitted.

    This method creates a command, possibly null, that is run on the remote prior to, and independent of, the actual simulation run. This operation is both machine– and simulator–specific, and simulators that use this Stage can call machine methods to get the string. ***Simulator*** declares abstract method `preRunModelProcPhaseP()`, which for no–model classes is a no–op in the concrete defintion. In the case of ***Sim_Neuron***, `preRunModelProcPhaseP()` calls `getCompileNeuronModelFilesStrPhaseP()`, declared as abstract by ***NeuronMachine*** (so that every subclass of ***NeuronMachine*** must implement it).

15. *Pre Run Model Processing During Submission (D)* (`preRunModelProcPhaseD()`) — Actions dealing with model file processing that must take place on the **remote** machine before the simulation is run, but that must be part of the actual job submission on the cluster (for example, on our SGE cluster, model file compilation cannot be done on the head node, and so must be done on a work node as part of the entire simulation job).

This method creates a string, possibly null, that goes into the run command that is buried in the job file. The string can be used to do model operations (such as compilation) as part of, and preceding, the actual simulation on the same node(s) that the simulation will use. This operation is both machine– and simulator–specific, and simulators that use this can call machine methods to get the string. In the case of Neuron, function is required, so ***NeuronMachine*** declares abstract method `getCompileNeuronModelFilesStrPhaseD()` that its every subclass must implement.

16. *Pre Run Job Processing* (`preRunJobProc()`) — Actions relating to simulation job submission that must be performed before the simulation job is run, such as the creation and positioning of a job file and the creation of a job submission shell script.

    Defined in ***Simulator***, this function uses `preRunCreateJobFile()` to create the job file. It's declared abstract in ***RunJobMachine*** and made concrete in the major job submission classes. There may be local differences, so the user may override `preRunCreateJobFile()` using subclasses to make the job file fit the situation without changing Core classes.

17. *Run Simulation* (`run()`) — Running the simulation submission shell script which includes (a) the previously compiled MATLAB executable, possibly preceded by (b) running a model compilation shell script. On job submission machines such as one that uses the Sun Grid Engine, this will involve the job file created in the *Pre Run Job Processing Stage* and the use of a job submission command such as `qsub`.

    Function `run()` is defined in ***Simulator*** and makes use of `runNoWait()` from the ***RunJobMachine*** class.

18. *Post Run Job Processing* (`postRunJobProc()`) — Actions relating to the run job that must be performed after the job has finished, such as moving job–submission byproducts to the simulation output directory for download. Rather than just throw these files away, having them accessible on the host makes troubleshooting much easier.

    The abstract declaration is in ***RunJobMachine***; the concrete definitions are in the different job submission classes.

19. *Pre Download* (`preDownload()`) — Actions that must be performed on the remote before the results files located in the simulation's output directory are downloaded, including compression of the output files.

    Defined and used in the ***Simulation*** class (actually called as part of `initiateDownload()` described next).

20. *Download* (`initiateDownload()`) — Asynchronous download of the simulation's compressed results file.

    Downloading in NeuroManager is asynchronous, typically involves on–the–fly shell script creation and the use of signal files, and is very host-dependent. `initiateDownload()` is defined in ***Simulation***, and called by the ***Simulator*** class, but the burden falls on `fileFromMachineNoWait()` in the ***TransferFileMachine*** class which creates a download shell script file. The script produces a signal file that the *Post Download Stage* detects and acts upon.

21. *Post Download* (`postDownloadProcessing()`) — Actions that must be performed on the host after the results files are downloaded, such as uncompressing and processing of results and metrics, or appending to a report, or adding results to a database.

Defined and used in the ***Simulation*** class. This stage also calls a simulator method called `postDownloadProcessingSimulatorSpecific()`, allowing for simulator–specific operations on simulation files on the host after download, such as copying a file to be attached to notifications (see the User Guide's description of the SineSim example).

22. *Post Simulation* (`postSimulation()`) — Actions that must be performed on the remote after the simulation download is complete; typically removal of simulation files and directories from the remote.
   Defined in the ***Simulation*** class and used by the ***Simulator*** class.
